# Expression of microRNAs 16, 20a, 150 and 155 in anal squamous intraepithelial lesions from high-risk groups

**DOI:** 10.1038/s41598-018-38378-6

**Published:** 2019-02-06

**Authors:** Andreia Albuquerque, Mara Fernandes, Oliver Stirrup, Ana Luísa Teixeira, Joana Santos, Marta Rodrigues, Elisabete Rios, Guilherme Macedo, Rui Medeiros

**Affiliations:** 1 Faculty of Medicine of the University of Porto, Porto, Portugal; 2 Gastroenterology Department, Centro Hospitalar São João, Porto, Portugal; 3 Molecular Oncology and Viral Pathology Group, Portuguese Oncology Institute of Porto Research Center (CI-IPOP), Portuguese Oncology Institute, Porto, Portugal; 4 Centre for Clinical Research in Infection and Sexual Heath, Institute for Global Health, University College London, London, UK; 5 Department of Pathology, Centro Hospitalar São João, Porto, Portugal; 6 Institute of Molecular Pathology and Immunology of the University of Porto (IPATIMUP) and i3S - Instituto de Investigação e Inovação em Saúde, University of Porto, Porto, Portugal; 7 Research Department, Portuguese League Against Cancer, Porto, Portugal; 8 CEBIMED, Faculty of Health Sciences, Fernando Pessoa University, Porto, Portugal

## Abstract

Anal squamous intraepithelial lesions (ASIL) or anal intraepithelial neoplasia (AIN) are precancerous lesions. microRNAs (miRNAs) have been implicated in cervical carcinogenesis, but have never been assessed in anal precancerous lesions. Our aim was to evaluate the expression of miR-16, miR-20a, miR-150 and miR-155 in several grades of ASIL obtained from high-risk patients, submitted to anal cancer screening from July 2016 to January 2017. Lesions were classified according to the Lower Anogenital Squamous Terminology (LAST) in low-grade (LSIL) and high-grade squamous intraepithelial lesions (HSIL), and the AIN classification in AIN1, AIN2 and AIN3. A hundred and five biopsies were obtained from 60 patients. Ten samples were negative (9.5%), 63 were LSIL (60%) and 32 were HSIL (30.5%) according to the LAST. Twenty seven (26%) were negative for dysplasia, 46 were classified as AIN1 (44%), 14 as AIN2 (13%) and 18 as AIN3 (17%) according to the AIN classification. There was no statistically significant difference in the fold expression of miR-16, miR-20a, miR-150 and miR-155, according to either classification. Although non- significant, there was an increasing trend in the miR-155 fold expression from negative samples to HSIL, with the highest fold expression increase in both LSIL and HSIL compared to the other miRNAs.

## Introduction

Anal squamous cell carcinoma (SCC) is strongly associated with anal human papillomavirus (HPV) infection, which has been observed to be present in nearly 90% of the cases^1^. The prevalence has been increasing in recent years in many populations, especially in Northern and Western Europe, America and Australia^2^, and it is expected to continue to grow in the coming decades^3,4^.

The terms anal squamous intraepithelial lesions (ASIL) or anal intraepithelial neoplasia (AIN) are used to describe anal SCC premalignant lesions. The Lower Anogenital Squamous Terminology (LAST) published in 2012, recommended that anogenital squamous intraepithelial lesions be classified as either low-grade squamous intraepithelial lesions (LSIL) or high-grade squamous intraepithelial lesions (HSIL) in all anogenital sites^5^. This classification replaced the former 3-tiered system that in the anal canal included the terms AIN1 (mild dysplasia) as low-grade lesions, AIN2 (moderate dysplasia) and AIN3 (severe dysplasia) as high-grade lesions. This differentiation between low and high-grade lesions is relevant for treatment indication, prognosis and follow-up. High-grade squamous intraepithelial lesions have a higher progression rate for cancer and treatment is normally advocated. Biomarkers can have an important role in the classification of anogenital squamous intraepithelial lesions^5^, by reducing inter- and intraobserver variability^6,7^. Currently, only p16 immunohistochemistry is recommended in specific cases, such as -IN2 cases (−IN2/p16 negative considered low-grade and −IN2/p16 positive as high-grade lesions)^5^. p16 is the best performing biomarker for classification currently available, but is not ideal^5^, with the possibility of false positives (7% of all anal LSIL will be p16 positive) and subsequent overtreatment, or false negative results and subsequent undertreatment (24% of AIN2 and 10% of AIN3 will be p16 negative)^8^.

microRNAs (miRNAs) are noncoding RNAs, approximately 21–23 nucleotides in length that have been studied and implicated in several types of cancers, acting as tumour suppressors or oncogenes (oncomirs)^9^. Research involving miRNAs may provide insight into HPV-related carcinogenesis and possible new biomarkers for cancer diagnosis, and determination of prognosis and optimal therapy^9^. Several studies have implicated multiple miRNAs in key pathways linked to cervical cancer, such as cell proliferation, apoptosis, migration and invasion^10^. Many of these studies compared miRNA expression in cervical SCC with that in normal cervical mucosa^10^. There are also studies that have evaluated the expression in cervical precancerous lesions, mostly with relatively small sample sizes^10^.

Anal and cervical carcinogenesis are considered to be very similar HPV-driven processes, although important differences exist. The incidence rate is much higher for cervical cancer, and there is a lower progression rate from anal high-grade lesions to cancer^11^. There are specific high-risk groups for HPV-related anal lesions, namely HIV-positive patients, especially those who are men who have sex with men (MSM)^11,12^, solid organ transplant recipients^13–15^ and women with a previous history of genital neoplasia^16–19^. Information on several aspects of anal carcinogenesis is still scarce, and much of our understanding and the approaches used for investigation have drawn on our knowledge of the cervix.

As far as we know, miRNAs have not previously been assessed in ASIL. The aim of this study was to evaluate the expression of miR-16, miR-20a, miR-150 and miR-155 in several histological grades of ASIL, obtained from high-risk patients. This miRNA panel was chosen based on published data related to cervical carcinogenesis, HPV infection and cell cycle influence^10^.

## Results

In total, 105 biopsies were obtained from 60 patients with a mean age of 42 ± 13 years. Fifty-three patients were HIV-positive (88%), 51 patients were men (85%), all men were men who have sex with men, and six of the nine women included had a previous history of genital neoplasia (67%). Two patients were on immunosuppressive drugs (3%), both were women also with a previous history of genital neoplasia. HPV 16 anal infection was detected in 28 patients (47%), HPV 18 in 18 patients (30%) and HR-HPV other than HPV 16/18 (but not excluding patients with HPV 16/18 coinfection) in 49 patients (82%), Table 1.

**Table 1:** Overall data of the patients (n = 60) and samples included in this study (n = 105).

Parameter	Value
**Patients Features**	
Men, n (%)	51 (85)
MSM, n (%)	51/51 (100)
Female (%)	9 (15)
Women with previous history of genital neoplasia, n (%)	6/9 (67)
Age (years), mean ± SD	42 ± 13
HIV-positive, n (%)	53 (88)
CD4 (cells/mm^3^) sample collection, mean ± SD	628 ± 278
Previous history of anal squamous cell carcinoma, n (%)	1 (2)
Pharmacologically immunosuppression, n (%)	2 (3)
Current or past smoking history, n (%)	19/45 (42)
Unknown history of smoking (missing data)	15/60 (25)
Anal HPV 16 positivity, n (%)	28 (47)
Anal HPV 18 positivity, n (%)	18 (30)
Other anal high-risk HPV*, n (%)	49 (82)
HPV negative/low-risk, n (%)	7 (12)
**Samples Features**	
LAST Classification	
Negative, n (%)	10 (9.5)
LSIL, n (%)	63 (60)
HSIL, n (%)	32 (30.5)
AIN Classification	
Negative for dysplasia, n (%)	27 (26)
AIN1, n (%)	46 (44)
AIN2, n (%)	14 (13)
AIN3, n (%)	18 (17)
Samples Location	
Anal, n (%)	85 (81)
Perianal, n (%)	20 (19)

AIN: anal intraepithelial neoplasia; HPV: human papillomavirus; HSIL: high-grade squamous intraepithelial lesions; LAST: lower anogenital tract terminology; LSIL: low-grade squamous intraepithelial lesions; MSM: men who have sex with men.

*High-risk HPV other than HPV16/18 (but not excluding patients with HPV 16/18 coinfection).

Ten samples were negative (9.5%), 63 were classified as LSIL (60%) and 32 as HSIL (30.5%) according to the LAST, with 95 ASIL in total. When considering the AIN classification, 27 (26%) were negative for dysplasia, 46 were classified as AIN1 (44%), 14 as AIN2 (13%) and 18 as AIN3 (17%), with a total of 78 AIN samples. Of the biopsies, 85 were anal (81%) and 20 were perianal (19%), Table 1.

Of the 60 patients included, there were 26 patients (43%) in whom biopsies were performed for more than one anal/perianal area, targeting different lesions. Information on the histological classification of the samples according to the LAST and AIN classification per patient are presented in Supplementary Tables S1 and S2, respectively.

**Figure 1:**
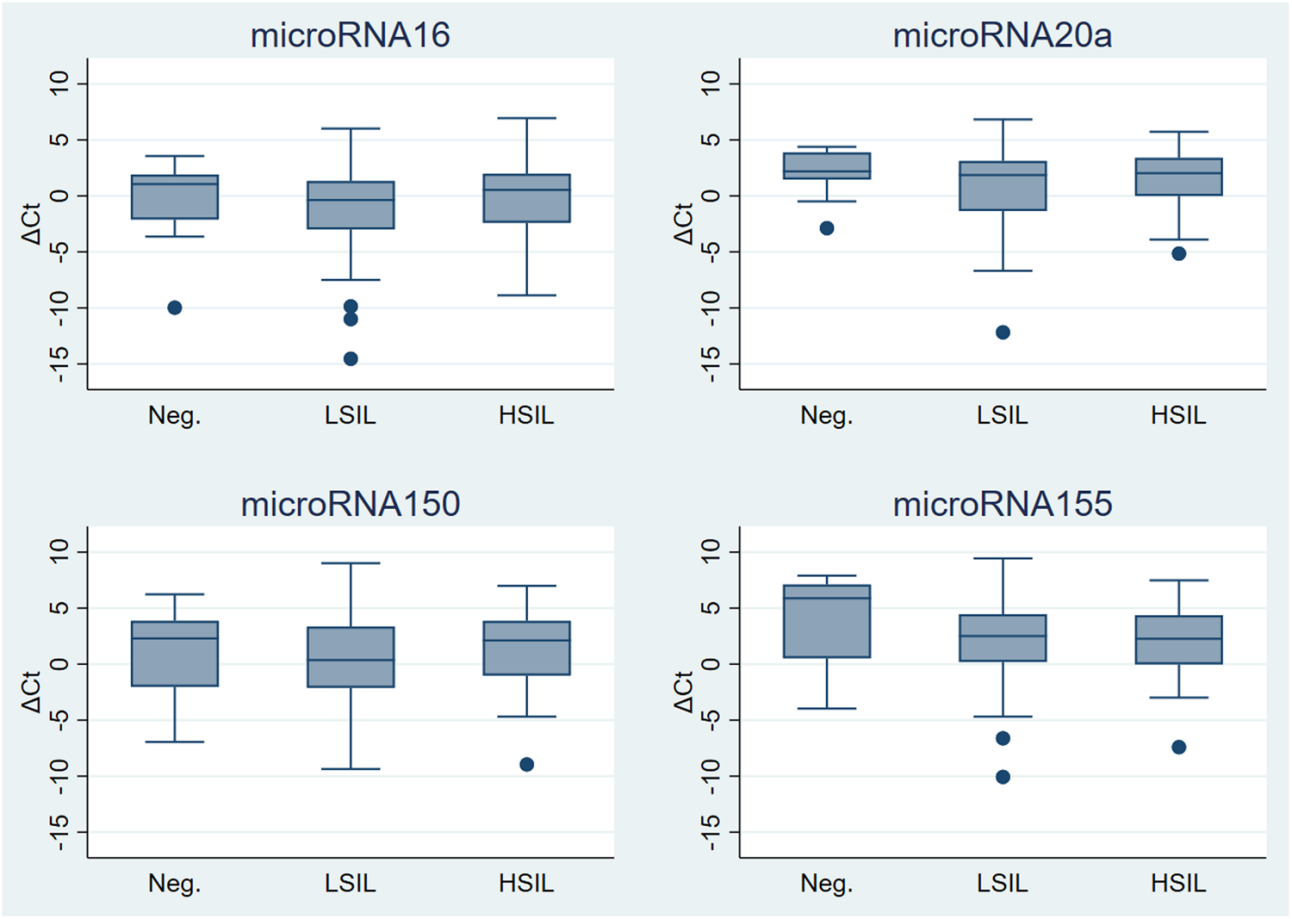
Boxplots of ΔCt values for each microRNA according to LAST classification of sample. HSIL: high-grade squamous intraepithelial lesions; LAST: lower anogenital tract terminology; LSIL: low-grade squamous intraepithelial lesions. Lower and upper limits of boxes represent lower and upper quartiles, respectively, the internal line is the median and the ‘whiskers’ show the range. Observations below ‘lower quartile −1.5* interquartile range (IQR)’ or above ‘upper quartile +1.5* IQR’ are not included in the range but are plotted individually (●).

There was no statistically significant difference in the fold expression of miR-16, miR-20a, miR-150 and miR-155 for anal/perianal LSIL and HSIL according to LAST, although an increasing trend in the fold expression was seen from negative to HSIL for miR-155. The highest fold expression increase in both LSIL and HSIL samples were seen for miR-155, (Table 2). Boxplots of ΔCt values for each miRNA according to LAST classification can be seen in the Fig. 1.

**Table 2:** Mean ΔCt and ΔΔCt values according to LAST and AIN classifications estimated using linear mixed effects models. ΔΔCt and P-values are displayed for comparison relative to reference group.

	ΔCt (95% CI)	ΔΔCt (95% CI)	2^−ΔΔCt^ (95% CI)	P-value*
***LAST classification***				
*microRNA20a*				
Negative	1.7 (−0.47 to 3.88)			0.814
LSIL	0.94 (0.03 to 1.85)	−0.76 (−3.08 to 1.55)	1.7 (0.34 to 8.44)	0.518
HSIL	1.04 (−0.19 to 2.26)	−0.67 (−3.12 to 1.78)	1.59 (0.29 to 8.68)	0.593
*microRNA150*				
Negative	0.72 (−1.79 to 3.24)			0.874
LSIL	0.74 (−0.34 to 1.82)	0.02 (−2.64 to 2.68)	0.99 (0.16 to 6.24)	0.990
HSIL	0.3 (−1.12 to 1.72)	−0.42 (−3.23 to 2.38)	1.34 (0.19 to 9.38)	0.768
*microRNA155*				
Negative	3.53 (1.22 to 5.84)			0.321
LSIL	2.19 (1.23 to 3.15)	−1.34 (−3.81 to 1.13)	2.53 (0.46 to 14.03)	0.287
HSIL	1.54 (0.23 to 2.84)	−1.99 (−4.61 to 0.62)	3.98 (0.65 to 24.41)	0.135
*microRNA16*				
Negative	−0.92 (−3.54 to 1.7)			0.930
LSIL	−1.02 (−2.1 to 0.07)	−0.1 (−2.9 to 2.7)	1.07 (0.15 to 7.45)	0.945
HSIL	−0.68 (−2.15 to 0.8)	0.24 (−2.72 to 3.2)	0.85 (0.11 to 6.61)	0.874
***AIN classification***				
*microRNA20a*				
No dysplasia	1.16 (−0.12 to 2.44)			0.309
AIN1	0.94 (−0.11 to 1.99)	−0.22 (−1.78 to 1.34)	1.16 (0.39 to 3.44)	0.785
AIN2	−0.21 (−1.94 to 1.53)	−1.37 (−3.44 to 0.71)	2.58 (0.61 to 10.87)	0.196
AIN3	1.96 (0.39 to 3.53)	0.8 (−1.14 to 2.74)	0.57 (0.15 to 2.21)	0.419
*microRNA150*				
No dysplasia	0.97 (−0.51 to 2.45)			0.461
AIN1	0.57 (−0.66 to 1.8)	−0.39 (−2.17 to 1.38)	1.31 (0.38 to 4.5)	0.664
AIN2	−0.77 (−2.75 to 1.2)	−1.74 (−4.08 to 0.6)	3.34 (0.66 to 16.88)	0.145
AIN3	1.11 (−0.69 to 2.92)	0.15 (−2.05 to 2.35)	0.9 (0.2 to 4.15)	0.895
*microRNA155*				
No dysplasia	2.64 (1.26 to 4.03)			0.612
AIN1	2.19 (1.06 to 3.31)	−0.46 (−2.16 to 1.25)	1.37 (0.42 to 4.48)	0.601
AIN2	1.07 (−0.82 to 2.97)	−1.57 (−3.85 to 0.71)	2.97 (0.61 to 14.39)	0.177
AIN3	1.86 (0.15 to 3.56)	−0.79 (−2.91 to 1.34)	1.73 (0.4 to 7.53)	0.468
*microRNA16*				
No dysplasia	−0.37 (−1.92 to 1.18)			0.606
AIN1	−1.42 (−2.68 to −0.17)	−1.05 (−2.97 to 0.87)	2.07 (0.55 to 7.85)	0.282
AIN2	−1.29 (−3.42 to 0.84)	−0.92 (−3.49 to 1.65)	1.89 (0.32 to 11.22)	0.483
AIN3	−0.21 (−2.12 to 1.7)	0.16 (−2.23 to 2.55)	0.9 (0.17 to 4.69)	0.896

*P-value in top row for each microRNA is likelihood ratio test for differences across all histological grades for each comparison, other P-values are calculated by Wald test for each grade in fitted model.

AIN: anal intraepithelial neoplasia; CI: confidence interval; HSIL: high-grade squamous intraepithelial lesions; LAST: lower anogenital tract terminology; LSIL: low-grade squamous intraepithelial lesions.

**Figure 2:**
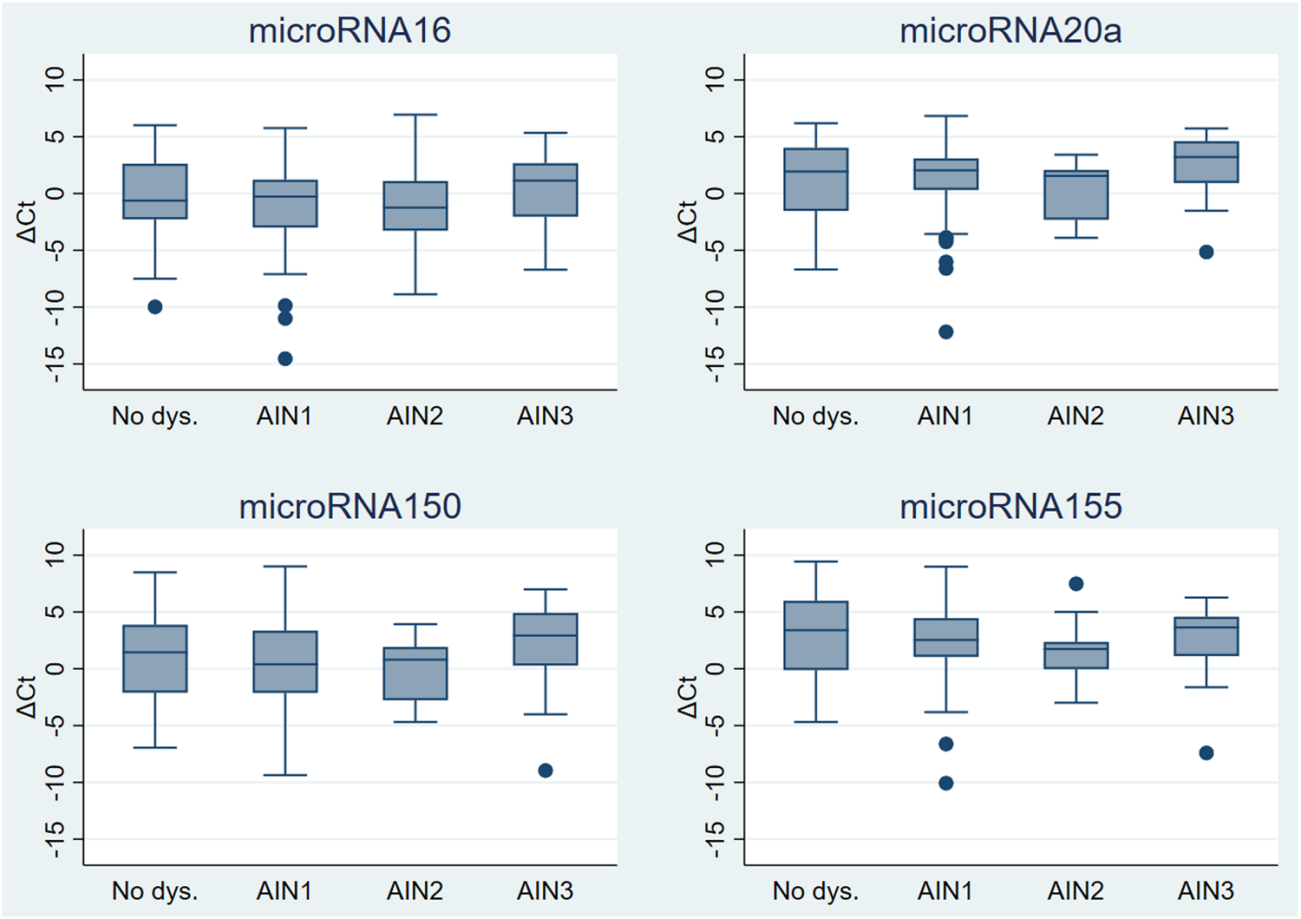
Boxplots of ΔCt values for each microRNA according to AIN classification of sample. AIN: anal intraepithelial neoplasia. Lower and upper limits of boxes represent lower and upper quartiles, respectively, the internal line is the median and the ‘whiskers’ show the range. Observations below ‘lower quartile −1.5* interquartile range (IQR)’ or above ‘upper quartile +1.5* IQR’ are not included in the range but are plotted individually (●).

There was also no statistically significant difference in the fold expression of miR-16, miR-20a, miR-150 and miR-155 for AIN1, AIN2 and AIN3 according to AIN classification. AIN2 samples showed the highest level of expression for miR-20a, miR-150 and miR-155, the highest difference was observed for miR-150 (Table 2). Boxplots of ΔCt values for each miRNA according to AIN classification can be seen in the Fig. 2.

There was no statistically significant different change in expression of these miRNAs according to the histological grade when analyses were adjusted for age, anal HPV genotype, lesion location, sex, HIV-positivity, smoking status or history of previous genital neoplasia (data not shown). There was no statistically significant difference in expression of these miRNAs according to HIV status or presence of high-risk HPV when these variables were used to adjust estimates for lesion classification or when analysed alone.

## Discussion

There are several studies showing an up-regulation of miR-16^20–23^, miR-20a^24–27^, miR-150^28,29^ and miR-155^21–23,30^ in cervical SCC (in comparison to normal mucosa). The expression of miRNA-20a^24,25,31^ and miRNA-150^28,29^ was also found to be associated with a worse cervical cancer prognosis. The miR-20a promotes migration and invasion by regulating tankyrase, TRF1-interacting ankyrin-related ADP-ribose polymerase 2 (TNKS2) expression in human cervical SCC cells^27^. TNKS2 overexpression can increase telomere length, acting as an oncogene^32,33^. The miR-150 modulates FOXO4 expression, resulting in cervical cancer cell growth and survival^28^.

The miR-16 has previously been recognized as a tumor-suppressive miRNA^10,34^, with decreased expression in several different cancers, but not in cervical cancer^10^. In cervical intraepithelial lesions (CIN), Wang *et al*.^20^ found an increasing trend of expression in CIN3, although non-statistically significant, when comparing normal (n = 38), CIN1/2 (n = 13) and CIN3 samples (n = 39). The miR-20a is part of the miR-17-92 cluster, and in one study it was shown that it was upregulated in the serum of CIN patients compared with those from the healthy controls^35^. In a study by Wilting *et al*.^21^, miR-150 expression was higher in CIN 2/3 samples (n = 18) *vs*. normal cervical samples (n = 10), although this result needs to be interpreted with caution due to the small sample size.

The miR-155 is a recognized oncomiR, promoting cervical cancer cell proliferation through suppression of LKB1 (tumor suppressor in cervical cancer)^30^. Two studies^21,36^ evaluating the expression in CIN2/3 samples *vs*. normal samples, failed to show a statistically significant higher expression in CIN2/3 samples, although there was an increasing trend in CIN2/3. miR-155 results in cervical studies/CIN samples are similar to this study: when comparing anal normal (n = 10) and anal HSIL samples (n = 32), although there was no statistically significant difference, a higher fold expression was seen in HSIL.

Using the AIN classification, the highest fold expression of miR-20a, miR-150 and miR-155 was seen in AIN2 samples, although this was non-statistically significant. In cervical studies involving these miRNAs, CIN2 samples were not analysed separately, so previous information regarding this is not available. We cannot rule out large average differences in expression between histological grades, but the observed distributions of values in individual samples clearly overlap greatly across histological grades indicating a lack of clear-cut differentiation.

The maximum number of CIN samples that have been previously tested for any of these four miRNAs in a single study was 52 samples (in this case for miR-16)^20^. As far as we know, the present study included the largest number of anogenital precancerous lesions tested for miRNA expression^10^, and histology was described according to both the AIN and the LAST classifications (in previous cervical studies only a single classification was used). The former AIN/CIN classification is still widely used, especially in Europe. An association between several (risk) factors and the fold expression of miRNAs was also conducted to understand how these factors could possibly influence the expression in histological grades.

Data from previous cervical studies provided important guidance for the choice of our miRNA panel, given the similarities between the two HPV-driven carcinogenic processes. In both cases, HPV is recognized as the major etiologic agent, there is a similar more susceptible histological area (squamocolumnar junction) and the same type of precancerous lesions (CIN/AIN). Most of the research in HPV-linked anogenital disease is focused on the cervix, with findings then commonly generalized to the anal canal. These generalizations are impaired by the fact that the cervix is by far the anatomical region most commonly affected by HPV-related lesions, and the progression rate of high-grade lesions in the cervix is around 1/80 per year *vs*. 1/377 per year in the anal canal (HIV-positive MSM in the highly active antiretroviral therapy era)^11^. Anal SCC is a largely HPV-driven disease (mainly HPV16), involving high-risk groups, with a very low prevalence in the general population, as for HSIL/AIN3 (also associated with HPV 16)^37^. These known differences, and the expected increase in incidence of anal SCC^3,4^ justify specific studies involving the anus.

The expression of these miRNAs in CIN samples showed, in most cases, an increasing trend in more severe grades (although non-statistically significant)^20,21,36^. Further studies with a large number of precancerous samples, including several grades of lesions, are important to clarify any possible association.

A large majority of patients included were HIV-positive MSM because this is the population with highest risk for anal SCC, and in which anal cancer screening has been recommended^38^. There have been several studies evaluating the involvement of cellular miRNAs during HIV infection, and as a potential biomarker in these populations^39–42^. One study showed that HIV-infected individuals with low or undetectable viral load exhibit a gene expression profile very similar to control or uninfected subjects^41^. Another study, analysing miRNA-150 (“anti-HIV miRNA”) levels in the peripheral blood of mononuclear cells of HIV-positive patients revealed that they are restored after highly active antiretroviral therapy, with no difference also shown for miRNA-16 levels according to HIV status/therapy^42^. There is no indication for providing anal cancer screening in healthy populations, so we do not have data/anal samples in low-risk controls/healthy individuals. Our HIV-positive cohort was homogeneous, with all patients well controlled on highly active antiretroviral therapy, so an effect of HIV status on the anal expression of miRNAs seems unlikely (with a different expression in high-risk patients relative to normal low-risk controls).

There are some limitations to be considered, although the number of anal samples included, for some comparisons of histological grades such as AIN2, the samples size is small. For estimates of miRNA expression in abnormal tissue relative to normal samples the confidence intervals for estimates were wide, meaning that large average differences between groups cannot be completely ruled out. There was a small number of samples for which p16 immunostaining was performed (n = 19), so comparison of miRNA expression according to p16 results have not been presented. There were only two patients on pharmacological immunosuppression (both were women with a previous history of genital neoplasia) and one patient with a previous history of anal SCC, so the impact of these features in miRNA expression was not analysed. These three patients, although in a small number, fit our inclusion criteria of high-risk patients for anal SCC who underwent anal cancer screening, and so were included. There was no association between miRNA expression and HPV presence or HIV status, but the numbers of patients who were HPV and HIV-negative are small.

This is the first ever study evaluating the expression of miRNAs in ASIL. Our findings indicate that at present, miR-16, miR-20a, miR-150 and miR-155 expression cannot be considered as biomarkers for the histological classification/differentiation of these lesions. There is also no indication, that our data in the anus and the previous published data in cervix, might be largely different at this level. For miR-155, although not statistically significant, there was an increasing trend in fold expression from negative samples to HSIL. The highest fold expression increase in both anal LSIL and HSIL was seen for this miRNA, and future studies might explore this further.

## Methods

### Study design and study population

This was a cross-sectional study, with recruitment of a sample of high-risk patients followed for anal SCC screening, from July 2016 to January 2017, in the Proctology outpatient clinic of Gastroenterology Department of Centro Hospitalar S. João, Porto, Portugal. Both first screening visits and follow-up visits were considered. Inability to provide written consent was an exclusion criteria. Any possible case with a suspicious lesion for anal SCC was not considered for this study. Information regarding gender, age at sample collection, smoking history, HIV-positivity, sexual orientation in men, previous history of genital neoplasia, pharmacological immunosuppression and previous anal SCC history were recorded.

Informed verbal and written consent was obtained from all patients that accepted entering the study. This study was approved by the Health Ethics Committee of Centro Hospitalar S. João and was performed in accordance with the 1964 Declaration of Helsinki and its later amendments.

### High-resolution anoscopy and sample collection

All patients underwent high-resolution anoscopy and anal/perianal biopsies were collected during the routine patient assessment, under high-resolution anoscopy. This technique was performed using a Carl Zeiss® colposcope (Carl Zeiss, Oberkochen, Germany), with patients observed in the knee–chest position (all procedures were performed by A.A.) An anoscope was inserted, and anal and perianal assessment was carried out under magnification, with a colposcope. Initially this was done without staining, and then 5% acetic acid and Lugol’s solution were used. Biopsies were performed using a mini-Tischler punch-biopsy forceps. No local anaesthesia was necessary for anal biopsies, for perianal biopsies a 1% lidocaine buffered with 8.4% of sodium bicarbonate was used. Two fragments were obtained, one for histological assessment and one fragment was frozen at −80 °C for miRNA analysis. The number of biopsies performed in each patient was determined by the number of lesions seen. When biopsies were done in several anal/perianal areas in the same patient, they were always targeting different lesion locations and normally done in the same procedure.

An anal cytology sample, collected as part of the regular follow-up/screening of these patients, was used for HPV genotyping (the cytology results themselves were not included for this analysis). Anal cytology was performed using a sterile polyester swab (Thermo Fisher Scientific, Waltham, Massachusetts, USA), previously moistened with water, with the patients in the knee–chest position. The swab was inserted in the distal rectum and then slowly withdrawn with rotational movements over a period of 20 seconds. Samples were placed into PreservCyt ThinPrep® solution (Hologic UK, Crawley, UK).

### Histological analysis

Histological samples were analysed in the Pathology Department of Centro Hospitalar S. João in Porto, Portugal, by experienced Pathologists using the same protocol and with consensus discussion of all difficult or equivocal cases. For this study, two histological classifications were recorded. One was according to the AIN classification (three-tiered nomenclature), using the presence/absence and grade of dysplasia: AIN1 (mild dysplasia), AIN2 (moderate dysplasia) and AIN3 (severe dysplasia). The other classification was according to the LAST^5^ two-tiered nomenclature: LSIL and HSIL. p16 immunohistochemistry was evaluated in equivocal cases as recommended by LAST guidelines, and p16-positive lesions were considered HSIL and p16-negative as LSIL^5^.

Negative biopsies obtained from the same group of high-risk patients were included in the analyses for comparison. The definition for negative anal biopsies is different in the two classifications and results were analysed accordingly. Anal biopsies with normal mucosa/reactive changes and non-dysplastic ASIL (including condylomas) were considered “negative for dysplasia”, according to the AIN classification. According to the LAST^5^ only anal biopsies with normal mucosa/reactive changes were considered “negative” since the sheer presence of a cytopathic effect of HPV (koilocytosis) and condyloma, even without dysplasia, are considered LSIL (koilocytosis and condyloma are usually a non-dysplastic ASIL).

### HPV genotyping

For this analysis, the remaining sample of the liquid-based anal cytology specimen that was collected during the normal patient assessment was used. HPV genotyping was performed using the cobas® HPV test. The test simultaneously provides pooled results for 12 high-risk (HR) genotypes (HPV 31, 33, 35, 39, 45, 51, 52, 56, 58, 59, 66, and 68) and individual results for HPV 16 and HPV 18. A negative result indicates either the absence of any HPV or the presence of only low-risk HPV infection (HPV 6 and 11). This analysis was conducted in the Pathology Department of Centro Hospitalar S. João in Porto, Portugal.

The procedure was performed following the manufacturer’s instructions. Initially, the DNA extraction (HPV nucleic acids and the control β-globin DNA) was carried out using the fully automated cobas x 480 instrument. The cobas z 480 analyzer was then used for real-time polymerase chain reaction (PCR) amplification of HR-HPV and a β-globin DNA. The interpretation of the results was accomplished using the software provided with the cobas z 480 analyzer.

### microRNA analysis

This analysis was conducted in the Portuguese Oncology Institute of Porto Research Center (CI-IPOP). The expression of four target miRNAs, miR-16, miR-20a, miR-150 and miR-155 and endogenous controls (RNU-44 and RNU-48) were assessed using TaqMan® technology. An investigator blinded to the histological grade of the lesions carried out the miRNA assays (M.F.).

Briefly, the tissue samples were homogenized and macerated into TripleXtractor reagent (Grisp- research solutions®) and using a syringe and 21 g needle. For miRNA isolation, the total RNA fraction was first isolated with a chloroform solution (Merck®) according Santos *et al*.^42^ protocol and for the purification we used the commercial kit GRS microRNA Kit (Grisp- research solutions®) after adjustments^43,44^.

The miRNA samples were then used as templates for cDNA synthesis using a Taqman®MicroRNA Reverse Transcription kit (Applied Biosystems®) and sequence specific stem-loop primers for hsa-miR-16, hsa-miR-20a, hsa-miR-150, hsa-miR-155, RNU-44 and RNU-48.

Based on a literature search encompassing studies on miRNA normalization in the cervical tissue, we selected RNU44 and RNU48 as endogenous candidate reference genes^22,45,46^. RNU-48 was used as an endogenous control for data normalization since it presented a stable expression pattern, meaning low mean delta threshold cycle values and standard deviation variations.

The thermal conditions were 16 °C for 30 minutes, followed by 42 °C for 60 minutes and 85 °C for 10 minutes as mention in Dias *et al*.^43^. The expression levels were analyzed by relative quantitative real-time PCR using a StepOne™qPCR Real-Time PCR machine. The reaction containing 1 × TaqMan® Fast Advanced Master Mix (Thermo Fisher Scientific®), 1 × probes (TaqMan® microRNA Expression Assays, miR-16: 000391; miR-150: 000473; miR-155: 002623; miR-20a: 000580; RNU-44: 001094; and RNU-48: 001006, Thermo Fisher Scientific®) and cDNA sample (~50 ng). Two technical replicates were made for each sample.

The amplification conditions were holding stage 95 °C for 20 seconds, followed by 45 cycles of 95 °C for 1 second and 60 °C for 20 seconds. Data analysis was performed using StepOne™Sofware v2.2 (Thermo Fisher Scientific®) with the same baseline and threshold set for each plate, in order to generate quantification cycle values for all the miRNAs in each sample.

### Outcomes

The primary outcome was to evaluate the expression of miR-16, miR-20a, miR-150 and miR-155 according to histological grade of the ASIL, using both the LAST and AIN classification. The secondary outcome was to evaluate factors that can modify the fold expression of these miRNAs, according to the histological grading.

### Statistical analysis

Continuous variables were described as mean ± standard deviation and categorical variables were described as absolute and relative frequencies.

microRNAs expression was initially quantified as delta threshold cycle values (ΔCt), defined as Ct target miRNA minus Ct RNU48. The mean Ct value of a given miRNA from normal/negative anal biopsies served as a basal level to calculate the relative level of the miRNA detected in each type of lesion.

There were several lesions seen in some patients (one biopsy for each lesion was taken), so analyses were conducted using random intercept models with outcome variables on the ΔCt scale. For the primary analyses ‘negative’ biopsies were treated as the reference categories and the average difference in ΔCt was estimated for each level of the LAST and AIN classifications, respectively. The model for each classification therefore provided estimates of ΔΔCt for each category of lesion relative to the reference group. The estimated ΔΔCt values and their 95% confidence interval (CI) were transformed to obtain estimates of relative change in expression normalized to an endogenous reference (RNU48) (2^−ΔΔCt^)^47^ for interpretation. The 2^−ΔΔCt^ value for ‘negative’ biopsies was 1 by definition.

Analyses were conducted to evaluate expression of the miRNAs according to histological grade (both classifications) with adjustment for each of the following patient characteristics: age (linear effect), anal HR-HPV positivity (any of the 12 possible types detected), HPV 16 positivity, HPV 18 positivity, lesion location (anal or perianal), sex, HIV-positivity, smoking status and a history of previous genital neoplasia. These analyses were again conducted using univariable linear mixed models with a random intercept term for each patient, and included all samples.

Statistical analysis was performed using Stata, version 14.1 (StataCorp, College Station, TX, USA).

## Supplementary information

Supplementary information
