# Supplementary material for: Expression of microRNAs 16, 20a, 150 and 155 in anal squamous intraepithelial lesions from high-risk groups
Source: Sci Rep. 2019 Feb 6;9:1523. doi: 10.1038/s41598-018-38378-6 (PMC6365520; doi:10.1038/s41598-018-38378-6)
Supplement: Supplementary file 1 — Supplementary information [file 41598_2018_38378_MOESM1_ESM.docx]

**Expression of microRNAs 16, 20a, 150 and 155 in anal squamous intraepithelial lesions from high-risk groups.**

Andreia Albuquerque,^1,2^# Mara Fernandes,^3^ Oliver Stirrup,^4^* Ana Luísa Teixeira,^3^* Joana Santos,^3^* Marta Rodrigues,^5^ Elisabete Rios,^1,5,6^ Guilherme Macedo,^1,2^ Rui Medeiros^1,3,7,8^

1. Faculty of Medicine of the University of Porto, Porto, Portugal.
2. Gastroenterology Department, Centro Hospitalar São João, Porto, Portugal
3. Molecular Oncology and Viral Pathology Group, Portuguese Oncology Institute of Porto Research Center (CI-IPOP), Portuguese Oncology Institute, Porto, Portugal.
4. Centre for Clinical Research in Infection and Sexual Heath, Institute for Global Health, University College London, London, UK.
5. Department of Pathology, Centro Hospitalar São João, Porto, Portugal.
6. Institute of Molecular Pathology and Immunology of the University of Porto (IPATIMUP) and i3S - Instituto de Investigação e Inovação em Saúde, University of Porto, Porto, Portugal.
7. Research Department, Portuguese League Against Cancer, Porto, Portugal.
8. CEBIMED, Faculty of Health Sciences, Fernando Pessoa University, Porto, Portugal.

# Author for correspondence:

Andreia Albuquerque

Gastroenterologist, MD

Faculty of Medicine of the University of Porto

Alameda Professor Hernâni Monteiro

4200-319 Porto, Portugal

Phone: +351 22 551 3600

[a.albuquerque.dias@gmail.com](mailto:a.albuquerque.dias@gmail.com)

Patient inclusion and sample collection was done by the principal investigator (A.A.) when she was still affiliated to the Gastroenterology Department, Centro Hospitalar São João, Porto.

* Oliver Stirrup, Ana Luísa Teixeira, Joana Santos contributed equally to this study

Table S1: Histological classification of samples according to the LAST classification, distributed per patient (n=60).

|  | | **HISTOLOGICAL CLASSIFICATION ACCORDING TO LAST** | | | **TOTAL**  **SAMPLES** |
| --- | --- | --- | --- | --- | --- |
|  |  | **Negative** | **LSIL** | **HSIL** |  |
| **PATIENTS** | 1 | 0 | 4 | 0 | 4 |
|  | 2 | 0 | 0 | 2 | 2 |
|  | 3 | 0 | 0 | 3 | 3 |
|  | 4 | 0 | 1 | 1 | 2 |
|  | 5 | 0 | 0 | 1 | 1 |
|  | 6 | 0 | 1 | 0 | 1 |
|  | 7 | 2 | 1 | 0 | 3 |
|  | 8 | 0 | 2 | 3 | 5 |
|  | 9 | 0 | 1 | 0 | 1 |
|  | 10 | 0 | 1 | 0 | 1 |
|  | 12* | 0 | 0 | 1 | 1 |
|  | 13 | 0 | 1 | 0 | 1 |
|  | 14 | 0 | 1 | 0 | 1 |
|  | 15 | 0 | 0 | 1 | 1 |
|  | 16 | 0 | 0 | 1 | 1 |
|  | 17 | 0 | 0 | 1 | 1 |
|  | 18 | 0 | 1 | 3 | 4 |
|  | 19 | 0 | 0 | 1 | 1 |
|  | 20 | 0 | 1 | 0 | 1 |
|  | 21 | 0 | 0 | 1 | 1 |
|  | 22 | 0 | 1 | 1 | 2 |
|  | 23 | 0 | 1 | 0 | 1 |
|  | 24 | 0 | 1 | 0 | 1 |
|  | 25 | 0 | 0 | 1 | 1 |
|  | 26 | 0 | 2 | 0 | 2 |
|  | 27 | 0 | 3 | 0 | 3 |
|  | 28 | 1 | 0 | 0 | 1 |
|  | 29 | 0 | 1 | 2 | 3 |
|  | 30 | 2 | 0 | 0 | 2 |
|  | 31 | 0 | 1 | 1 | 2 |
|  | 32 | 0 | 1 | 0 | 1 |
|  | 33 | 0 | 1 | 0 | 1 |
|  | 34 | 0 | 1 | 0 | 1 |
|  | 35 | 1 | 0 | 0 | 1 |
|  | 36 | 0 | 1 | 2 | 3 |
|  | 37 | 0 | 2 | 0 | 2 |
|  | 38 | 0 | 1 | 0 | 1 |
|  | 39 | 0 | 1 | 0 | 1 |
|  | 40 | 0 | 2 | 0 | 2 |
|  | 41 | 1 | 0 | 2 | 3 |
|  | 42 | 0 | 1 | 1 | 2 |
|  | 43 | 0 | 1 | 0 | 1 |
|  | 44 | 0 | 2 | 0 | 2 |
|  | 45 | 2 | 0 | 0 | 2 |
|  | 46 | 0 | 1 | 0 | 1 |
|  | 47 | 0 | 1 | 0 | 1 |
|  | 48 | 0 | 1 | 0 | 1 |
|  | 49 | 0 | 4 | 0 | 4 |
|  | 50 | 0 | 1 | 0 | 1 |
|  | 51 | 0 | 4 | 0 | 4 |
|  | 52 | 0 | 1 | 0 | 1 |
|  | 53 | 1 | 1 | 0 | 2 |
|  | 54 | 0 | 1 | 1 | 2 |
|  | 55 | 0 | 0 | 1 | 1 |
|  | 56 | 0 | 4 | 0 | 4 |
|  | 57 | 0 | 0 | 1 | 1 |
|  | 58 | 0 | 1 | 0 | 1 |
|  | 59 | 0 | 1 | 0 | 1 |
|  | 60 | 0 | 1 | 0 | 1 |
|  | 61 | 0 | 2 | 0 | 2 |
| **Total** | | 10 | 63 | 32 | 105 |

* patient 11 does not exist

HSIL: high-grade squamous intraepithelial lesions, LSIL: low-grade squamous intraepitelial lesions, LAST: lower anogenital tract terminology.

Table S2: Histological classification of samples according to the AIN classification, distributed by patient (n=60).

|  | | **HISTOLOGICAL CLASSIFICATION ACCORDING TO AIN** | | | | **TOTAL**  **SAMPLES** |
| --- | --- | --- | --- | --- | --- | --- |
|  |  | **No dysplasia** | **AIN1** | **AIN2** | **AIN3** |  |
| **PATIENTS** | 1 | 0 | 4 | 0 | 0 | 4 |
|  | 2 | 0 | 0 | 2 | 0 | 2 |
|  | 3 | 0 | 0 | 0 | 3 | 3 |
|  | 4 | 1 | 0 | 1 | 0 | 2 |
|  | 5 | 0 | 0 | 0 | 1 | 1 |
|  | 6 | 1 | 0 | 0 | 0 | 1 |
|  | 7 | 2 | 1 | 0 | 0 | 3 |
|  | 8 | 2 | 0 | 0 | 3 | 5 |
|  | 9 | 0 | 1 | 0 | 0 | 1 |
|  | 10 | 0 | 1 | 0 | 0 | 1 |
|  | 12* | 0 | 0 | 0 | 1 | 1 |
|  | 13 | 0 | 1 | 0 | 0 | 1 |
|  | 14 | 1 | 0 | 0 | 0 | 1 |
|  | 15 | 0 | 0 | 1 | 0 | 1 |
|  | 16 | 0 | 0 | 0 | 1 | 1 |
|  | 17 | 0 | 0 | 0 | 1 | 1 |
|  | 18 | 0 | 1 | 2 | 1 | 4 |
|  | 19 | 0 | 0 | 1 | 0 | 1 |
|  | 20 | 0 | 1 | 0 | 0 | 1 |
|  | 21 | 0 | 0 | 0 | 1 | 1 |
|  | 22 | 0 | 1 | 0 | 1 | 2 |
|  | 23 | 1 | 0 | 0 | 0 | 1 |
|  | 24 | 0 | 1 | 0 | 0 | 1 |
|  | 25 | 0 | 0 | 0 | 1 | 1 |
|  | 26 | 2 | 0 | 0 | 0 | 2 |
|  | 27 | 0 | 3 | 0 | 0 | 3 |
|  | 28 | 1 | 0 | 0 | 0 | 1 |
|  | 29 | 0 | 1 | 1 | 1 | 3 |
|  | 30 | 2 | 0 | 0 | 0 | 2 |
|  | 31 | 0 | 1 | 1 | 0 | 2 |
|  | 32 | 1 | 0 | 0 | 0 | 1 |
|  | 33 | 0 | 1 | 0 | 0 | 1 |
|  | 34 | 0 | 1 | 0 | 0 | 1 |
|  | 35 | 1 | 0 | 0 | 0 | 1 |
|  | 36 | 0 | 1 | 1 | 1 | 3 |
|  | 37 | 0 | 2 | 0 | 0 | 2 |
|  | 38 | 0 | 1 | 0 | 0 | 1 |
|  | 39 | 1 | 0 | 0 | 0 | 1 |
|  | 40 | 0 | 2 | 0 | 0 | 2 |
|  | 41 | 1 | 0 | 2 | 0 | 3 |
|  | 42 | 0 | 1 | 0 | 1 | 2 |
|  | 43 | 1 | 0 | 0 | 0 | 1 |
|  | 44 | 0 | 2 | 0 | 0 | 2 |
|  | 45 | 2 | 0 | 0 | 0 | 2 |
|  | 46 | 0 | 1 | 0 | 0 | 1 |
|  | 47 | 1 | 0 | 0 | 0 | 1 |
|  | 48 | 0 | 1 | 0 | 0 | 1 |
|  | 49 | 0 | 4 | 0 | 0 | 4 |
|  | 50 | 0 | 1 | 0 | 0 | 1 |
|  | 51 | 2 | 2 | 0 | 0 | 4 |
|  | 52 | 0 | 1 | 0 | 0 | 1 |
|  | 53 | 1 | 1 | 0 | 0 | 2 |
|  | 54 | 0 | 1 | 1 | 0 | 2 |
|  | 55 | 0 | 0 | 0 | 1 | 1 |
|  | 56 | 1 | 3 | 0 | 0 | 4 |
|  | 57 | 0 | 0 | 1 | 0 | 1 |
|  | 58 | 1 | 0 | 0 | 0 | 1 |
|  | 59 | 1 | 0 | 0 | 0 | 1 |
|  | 60 | 0 | 1 | 0 | 0 | 1 |
|  | 61 | 0 | 2 | 0 | 0 | 2 |
| **Total** | | 27 | 46 | 14 | 18 | 105 |

* patient 11 does not exist

AIN: anal intraepithelial neoplasia.
